# Rural-urban land and food system changes in West Africa

**DOI:** 10.1016/j.isci.2025.114007

**Published:** 2025-11-12

**Authors:** Kira Fastner, Kofi Yeboah Asare, Andreas Buerkert

**Affiliations:** 1 Organic Plant Production and Agroecosystems Research in the Tropics and Subtropics, Organic Agricultural Sciences, University of Kassel, Witzenhausen, Germany; 2 School for Development Studies, University of Cape Coast, Cape Coast, Ghana

**Keywords:** Earth sciences, Remote sensing, Agricultural land, Food policy, Land use

## Abstract

West Africa’s land and food systems are increasingly reshaped by the effects of urbanization and trade liberalization. This study aims at quantifying changes in food production and consumption patterns along selected rural-urban transects in Niger and Ghana over the past 20 years. Remote sensing analyses of cropland changes were combined with data on food flows and household surveys. Our findings indicate that rapid urban growth and rural market integration in West Africa are driving distinct, location-specific transformations. Niger’s land and food systems are increasingly linked to major West African coastal cities through growing intra-regional trade. This is leading to the expansion of cropland and rising consumption of imported products. In rural and peri-urban Accra, Ghana, the ongoing conversion of cropland to built-up areas is challenging food access and security. To enhance food system resilience, multidirectional dependencies between cities and their nearby and distant rural hinterlands must be recognized and addressed.

## Highlights

• Combined analysis of cropland and food consumption changes along rural-urban transects
• Transformation processes differ across national and regional contexts
• Food system strategies should address rising (cross-border) rural-urban linkages

## Graphical abstract

**Figure:**
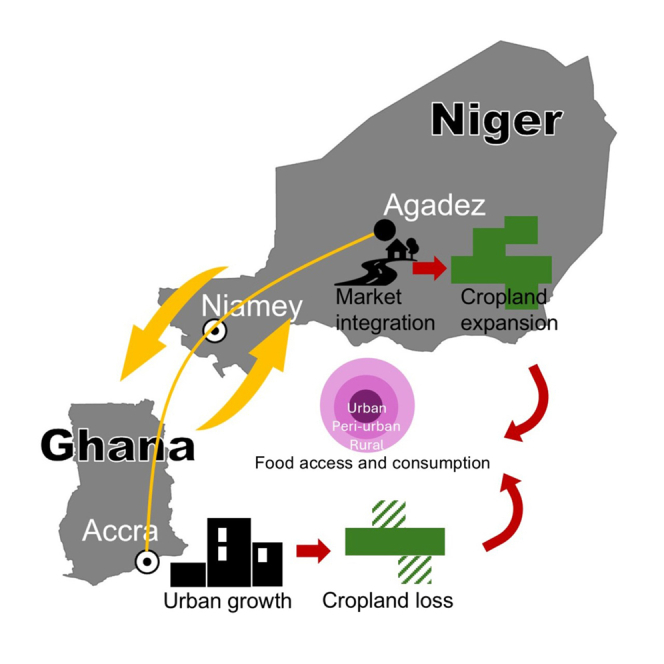

## Introduction

Between 2000 and 2030, West Africa’s urban population is projected to rise from 81 million to 278 million people^1^ accompanied by a doubling of the urban land area.^2^ The largest urban agglomerations are located in the (sub-) humid coastal countries, especially in Nigeria, Ghana, Ivory Coast, Benin, and Togo, but urban growth increasingly stretches inland to the (semi-) arid countries, including Mali and Niger.^3^^,^^4^ The expansion of urban built-up areas has overgrown former agricultural land in and around cities,^5^^,^^6^ and substantial areas of natural vegetation elsewhere have been converted into agricultural land^7^^,^^8^ to feed the rapidly growing urban population. Consequently, growing cities increasingly rely on food flows from more distant sources.^9^^,^^10^^,^^11^ Along with a broad shift in food consumption patterns over the past 50 years,^12^^,^^13^ food self-sufficiency of both urban and rural areas has declined. Recent data show rising import volumes of specific food items from the US, Europe, and Asia to West Africa, particularly of rice (*Oryza sativa* L.), wheat (*Triticum* L.), frozen chicken, and packaged foods.^14^^,^^15^ At the same time, the intra-regional trade (within West Africa) of perishable food items—such as fruits, vegetables, and animal products—is growing, likely at an even faster rate than of imported food products.^13^^,^^16^^,^^17^ These local high-value food products are increasingly sourced from West Africa’s rural hinterlands.^18^^,^^19^ The sale of food products to urban consumers with greater purchasing power has become an essential livelihood strategy for many rural smallholders.^18^^,^^19^ Rural market integration through improved road infrastructure contributes to intensified rural-urban linkages within West Africa and leads to complex and often opaque transnational food supply networks across more than 2,000 km.^11^^,^^18^

**Figure 1:**
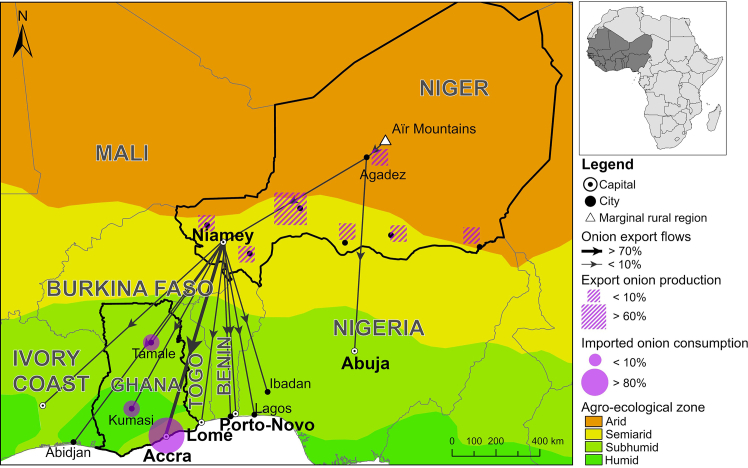
Onion flows from Niger to Ghana Cross-border onion flows from northern Niger to rapidly growing cities in West Africa^15^ across different agro-ecological zones.

Even though there have been recent investigations on how urbanization and food demand in West Africa can lead to agricultural production displacements,^8^ expanding foodsheds,^10^^,^^11^ and telecoupled agricultural production,^18^^,^^19^ only few attempts have been made to characterize and quantify social-ecological links between rural food “source” regions and, often spatially distant, urban “sink” regions. Differences in food access and consumption patterns along urban-rural gradients in West Africa, as well as the multidirectional dependencies over increasing distances, remain unexplored. In this study, we distinguish between urban, peri-urban, and rural cropland and food consumption shifts in the two West African countries of Niger and Ghana. The selected study locations are situated along the onion (*Allium cepa* L.) trading route from remote Agadez in northern Niger to its southern capital Niamey, and further to the Greater Accra Region of coastal Ghana. The selected locations exemplify how distant places and livelihoods are connected through new food supply chains (Figure 1).^18^

Agro-ecological conditions, urbanization rates, and income levels differ between the two countries. In northern Niger, limited rainfall (<200 mm year^−1^) during a short rainy season from July to September^20^ makes rainfed agricultural production impossible and limits the availability of groundwater for irrigated agricultural production. The comparatively longer rainy season in southern Niger from June to October and an average annual precipitation of 580 mm^21^ allow for rainfed agricultural production. The climate in Ghana is (sub-) humid with a bi-modally distributed, annual rainfall of 760 mm, and year-round temperatures averaging 27°C.^22^ In 2023, the gross domestic product (GDP) per capita in Ghana was 2,260 USD, around 3.5 times higher than that in Niger (643 USD^23^). In West Africa, Niger has the lowest share of people living in urban areas (around 20 %), whereas Ghana has the highest, with more than 50 % of its population residing in cities.^4^ However, with an annual population growth rate of around 3.3% in 2024, Niger has one of the highest population growth rates worldwide.^4^^,^^24^ Ghana’s population is growing at an annual rate of 1.9%.^24^ In Niger, more than 70% of the population is engaged in agricultural activities, while, in Ghana, around 40% remain working in the primary sector.^25^ In addition to international disparities, important differences on the regional scale exist.^26^^,^^27^ In both countries, peri-urban and rural communities remain highly dependent on farming for their livelihoods (>90% in Niger, 70% in Ghana), whereas urban populations are often engaged in the off-farm food economy, including food processing and marketing (50% in Niger, 70% in Ghana^28^). In both countries, localized competition for land between urban development, natural ecosystems, and agriculture is high.^5^^,^^29^^,^^30^

This study aims to analyze how cropland and food consumption patterns in rural-urban regions of Agadez, Niamey, and Accra have changed over the past 20 years. We hypothesize that urban growth and rural market integration (1) are correlated with changes in cropland size along the selected rural-urban transects and (2) affect rural, peri-urban, and urban food consumption patterns differently.

## Results

### Cropland changes

**Figure 2:**
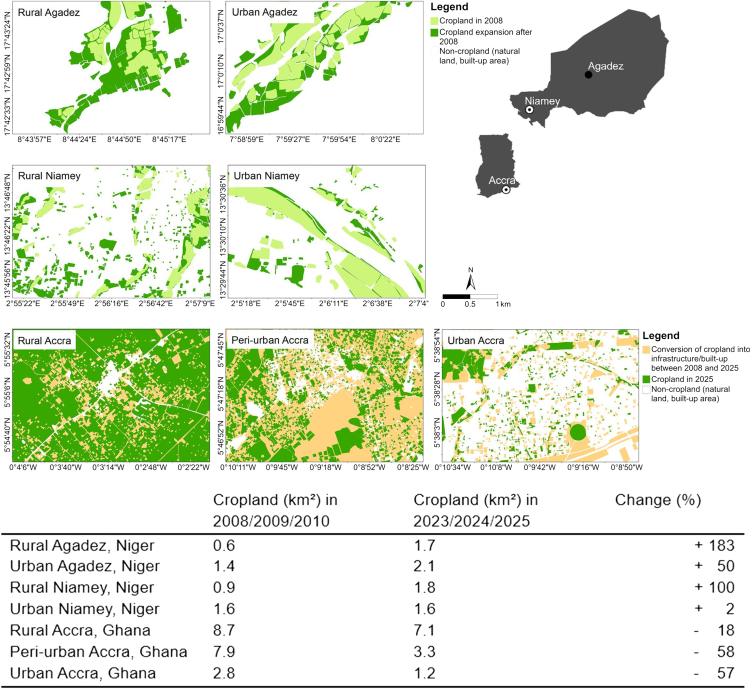
Visualized and calculated cropland changes in rural-urban Agadez, Niamey, and Ghana Classified Google Earth satellite images from 2008/2009/2010 and from 2023/2024/2025 in 50 km^2^ sections.

In all selected study locations in Niger, cropland expanded between 2008 and 2025 (Figure 2). As a result, formerly bare and natural land, with sparse vegetation, was increasingly converted to cultivated cropland. The expansion of cropland was particularly significant in the selected rural locations in Niger. In the rural area near Agadez, farmland grew by more than 180%, and near Niamey by 100%. The classification of fields based on visible field boundaries in the selected satellite images indicates that the sizes of individual fields have not increased. Agricultural land is largely managed by local smallholders, with no involvement of external investors or large agribusinesses. The average field size in rural Agadez was 0.6 ha in 2010 and remains so 15 years later. In rural Niamey, the average field size declined from 0.45 ha in 2010 to 0.24 ha in 2023.

In urban Agadez, many new agricultural field gardens were established within newly developed residential areas. Cultivated land has expanded by 50%, whereby average farm size declined from 2.1 to 1.7 ha between 2009 and 2025. The extent of cropland in urban Niamey has stagnated, even though population numbers and built-up areas have risen. Cropland along the Niger River has not been affected by the growth of built-up areas.

Contrarily, cropland in Accra, Ghana, has declined for all selected rural, peri-urban, and urban locations in a comparable time interval. In urban and peri-urban regions, cropland more than halved between 2008 and 2023. At the selected rural location, 18% of the agricultural land has been turned into built-up area. Many new residential areas have developed in areas of former agricultural smallholdings.

### National crop production, intra-regional, and international trade

**Figure 3:**
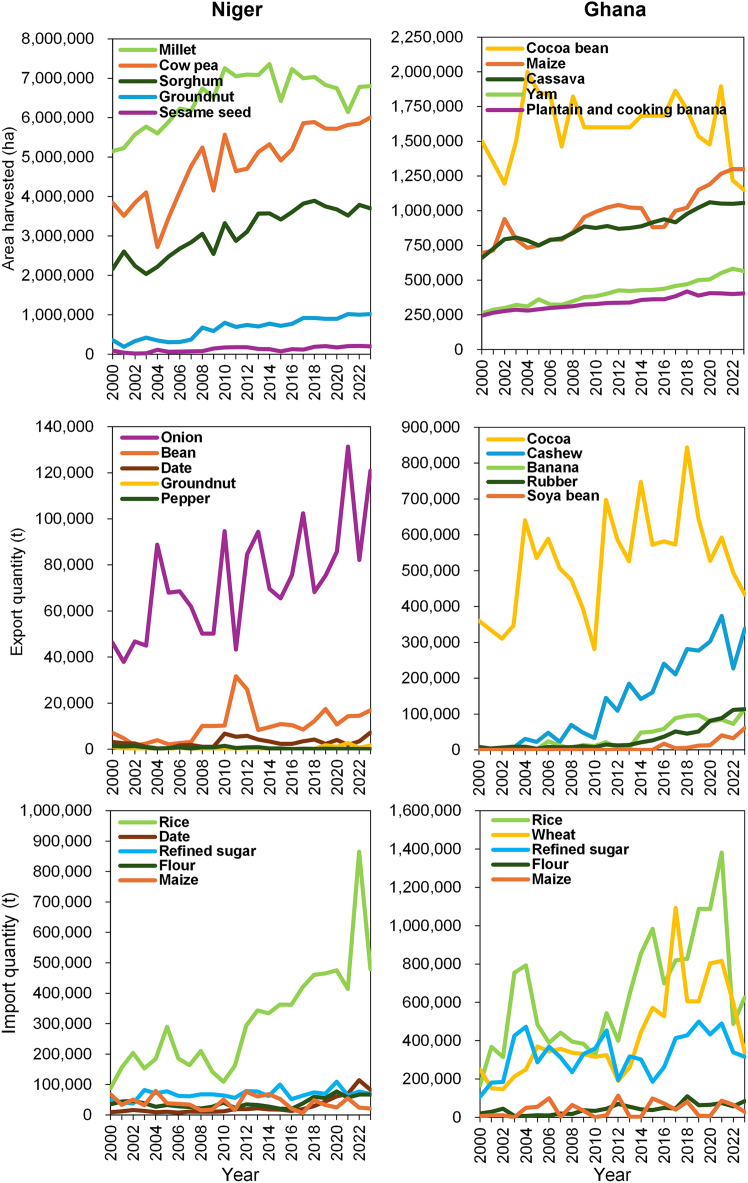
Total harvested areas (ha) of the five main cultivated crops in Niger and Ghana and import and export volumes of major non-animal products from 2000 to 2023 Data were retrieved from the FAOSTAT^15^ database.

The most recent FAOSTAT^15^ dataset shows that the majority of harvested cropland in Niger remains cultivated with millet (*Pennisetum glaucum* L.), cowpea (*Vigna unguiculata* (L.) Walp.), and sorghum (*Sorghum* Moench.). These crops are primarily produced for national consumption, with only minor shares traded intra-regionally. Between 2000 and 2023, the harvested millet area had an average annual growth rate of 1.2% while the harvested cowpea and sorghum areas grew by 2% and 2.4%, respectively (Figure 3). Niger’s main export cash crop is onion, which is traded intra-regionally. Its average annual export growth rate was 4.3% between 2000 and 2023. Niger mainly imports rice, whereby average annual rice imports grew by 7.7% between 2000 and 2023. Imports of other staple crops to Niger only slightly increased remaining at less than 100,000 tons per year.

Most of Ghana’s cropland area is dedicated to cocoa beans (*Theobroma cacao* L.), maize (*Zea mays* L.), and cassava (*Manihot esculenta* Crantz.). While maize and cassava are consumed nationally and traded intra-regionally, cocoa beans are mainly produced for global export markets. From 2000 to 2023, harvested areas of maize and cassava increased by 2.8% and 2.1% annually, while cocoa’s harvested area decreased on average by 1.1%, with strong fluctuations over time. Ghana’s most important export product is cocoa with an average annual export growth rate of 0.8%, but cashew (*Anacardium occidentale* L.) plays an increasingly important role in global trading with an average annual growth of 21.8% since the 2000s. Similar to Niger, Ghana mainly imports rice but also wheat from outside West Africa. Rice imports to Ghana grew by 5.9% between 2000 and 2023.

In both countries, the quantities of the main imported and exported products, except for cocoa, have grown at remarkable rates since the 2000s. This highlights the importance of both intra-regional and international trade for West African countries.

### Food access and consumption patterns along rural-urban transects

**Figure 4:**
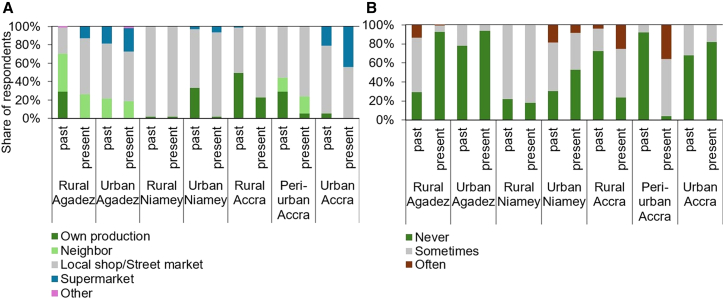
Food access points and perceptions of hunger in rural-urban locations of Niger and Ghana (A) Food access points and (B) perception of hunger (past-present) of the surveyed population.

Across locations, respondents to our survey reported a decline in subsistence-based food production over the past 20 years (Figure 4A). This means that overall dependence on food markets is rising, both in regions where cropland is being lost and in those where cropland has expanded. Today, most people at all study locations purchase their food in local shops and in open-air and street markets. The share of urban residents purchasing food in supermarkets has increased, while for rural and peri-urban populations supermarkets do not play a significant role in food access.

Participants were asked to estimate how their perception of hunger has changed over the past 20 years (Figure 4B). In rural Agadez, where cropland increased the most, a significant decline in hunger was reported. While 71% of the respondents reported having gone sometimes or often hungry in the past; today 7% often or sometimes face hunger. In urban Agadez, the share of respondents who often or sometimes face hunger declined from 22% to 6%. In rural Niamey, the share of respondents sometimes facing hunger has slightly increased from 78% to 82%, whereas in urban Niamey the hunger rate declined from 69% to 47%. Unlike all other locations, in rural and peri-urban Accra, the share of people experiencing hunger, either often or occasionally, has strongly risen. In rural Accra, the share has increased from 27% to 76%, and, in peri-urban Accra, it has surged from 8% to 96%. Among all surveyed locations, peri-urban Accra currently has the highest share of people who frequently go hungry (36%) and has also experienced the greatest cropland loss over the past 25 years.

Surveyed residents of peri-urban Accra reported that the rapid decline of cropland has forced many to seek employment in the off-farm food economy or low-paid jobs in the broader service sector, such as hairdressing, construction labor, or selling crafts. As a consequence, food must now be purchased from peri-urban food markets or sourced and transported from more distant urban markets. For low-income population groups, this increasingly challenges food access and security.

Changes in daily diets have been perceived differently for the selected rural-urban locations in Niger and Ghana (Table 1). In urban Agadez and urban Accra, only one factor has changed at each location: an increase in daily snack consumption in Agadez and more frequent consumption of milk products in Accra. At all other locations, at least two factors have changed. Especially in and around Niamey, several dietary aspects have been reported to differ compared to 20 years ago. In rural Niamey, a decline in daily meals and in the frequency of meat and milk product consumption has been noted. In urban Niamey, increases in daily snacks, in the share of vegetables in daily food consumption, and in the frequency of meat consumption have been reported.

**Table 1:** Changes in rural-urban food consumption patterns in Niger and Ghana.

	Number of daily meals	Number of daily snacks	Share of cereals in daily meals	Share of vegetables in daily meals	Share of fruits in daily meals	Frequency of meat consumption	Frequency of milk product consumption
Rural Agadez	0	+	0	0	0	+	–
Urban Agadez	0	+	0	0	0	0	0
Rural Niamey	–	0	–	+	0	–	–
Urban Niamey	0	+	0	+	0	+	–
Rural Accra	–	–	0	0	0	0	–
Peri-urban Accra	–	0	0	0	0	0	–
Urban Accra	0	0	0	0	0	0	+

Reported changes (>50% of respondents) in food consumption patterns over the past 20 years (0, no change; +, increase; −, decrease)

In the selected rural locations, close to the primary cities in Ghana and Niger, a decline in the number of daily meals from 3 to 2 was noted by the majority of the respondents. In urban Niger, snacks, such as cookies and other convenience products, are increasingly purchased. In rural Niamey, the share of cereals in daily diets decreased from more than 75% to 25%–50% for most respondents. At the same time, daily vegetable consumption increased from less than 25% to 25%–50%.

**Figure 5:**
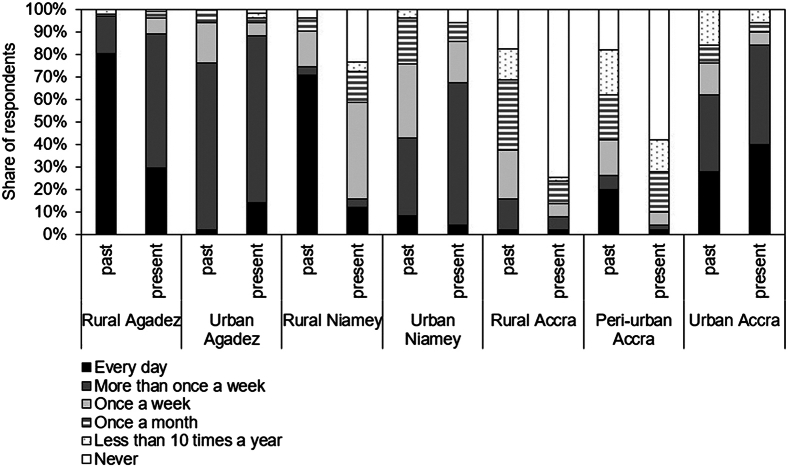
Frequency of milk product consumption (past-present) of the surveyed population in rural-urban locations of Niger and Ghana

Except for urban Agadez, at all study locations a change in the consumption of milk products (including milk powder) was recorded (Figure 5). While milk consumption increased among the urban residents in Agadez and Accra, the rural study locations experienced a significant decline. This trend was especially pronounced in rural Agadez and rural Niamey. In Agadez, the percentage of respondents consuming milk daily dropped from 80% to 29%, and in Niamey it declined from 71% to 12%. The main reason for this trend was a change in agricultural activities from livestock husbandry for subsistence or infrequent sale of animals to a focus on the production of marketable crops. With the profit from selling cash crops, milk powder is now occasionally purchased from the cities.

## Discussion

In this study, we first examined how cropland size is affected by urbanization and increasing market integration. Our findings show that cropland in and around Accra in Ghana’s humid coastal zone is shrinking, while it is expanding in parts of (semi-) arid Niger. These patterns are consistent with evidence of cropland losses in urbanizing regions in general^31^ and in Accra in particular.^32^ Only a few studies have examined broader land use changes in Niger. Recent case studies from northern Niger^18^^,^^19^ confirm that improved market access can lead to rapid cropland expansion despite the arid climate of the region. These different and likely interdependent dynamics in Ghana and Niger through intra-regional trade connections may have multiple underlying causes. Compared to Ghana, Niger’s society is still largely agrarian with a lower share of people residing in cities and a much less diversified economy.^28^ While Ghana produces cocoa and cashew nuts in demand on global markets, Niger lacks agricultural products with significant global trade potential.^15^ In Niger, the production of high-value cash crops for West African urban markets (particularly onion and potato) has opened new income opportunities for both rural and (peri-) urban households. This could be one explanation why farmland is expanding, even in urbanizing regions. Moreover, high population pressure likely causes an increase in the number of agricultural smallholders. Rising investments in the tapping of riverbed, groundwater, and fossil water sources allow the expansion of irrigated cropland to produce market crops for export to cities.^33^^,^^34^^,^^35^ While Ghana engages in global export, it appears that Niger is benefiting mainly from rising intra-regional food trade of high-value products. In Ghana, urban and peri-urban agriculture (UPA) faces multiple challenges, such as the expansion of urban built-up area, external investments in landholdings, and land grabbing, which causes land tenure insecurity.^5^^,^^36^ It has often been argued that UPA is a way to tackle food insecurity of the urban poor.^37^^,^^38^^,^^39^ However, practical constraints, poor urban governance, and policy failures hinder the maintenance and development of Ghana’s UPA activities.^40^ Rather than being self-sufficient, rapidly growing cities, like Accra, increasingly rely on other, more distant food sources. Intra-regional food trade across different agro-ecological zones has intensified, despite harsh climate conditions, political turmoil, and challenging transport infrastructure.^17^ This can lead to telecoupled land systems beyond national borders.^18^ While creating new income opportunities, the rapid expansion of cropland, such as in the rural Agadez region, may also have major negative ecological consequences, including increased pressure on scarce water resources.^18^ Climate change and unpredictable rainfall can lead to crop failures, severely affecting farmers who have specialized in the production of a single cash crop.^41^ The expansion of less diversified agricultural systems poses social-ecological risks and questions the sustainability of these transformation processes.^18^ Such possible consequences of rural market integration are insufficiently acknowledged in research and in policies, despite their importance for shaping sustainable land use and food systems.

Second, we aimed to show how changes in croplands influence food access and consumption patterns. Agricultural land use changes not only directly affect the income levels of households depending on farming activities but also indirectly shape diets through the availability of food, which is not purchased. Even though rural households, of which the majority depend on farming activities, purchase most of the food they consume,^42^ it appears that the consumption patterns of rural populations largely follow the transitions in agricultural land use. This assumption is supported by the rapid decline in milk consumption and the increase in the consumption of potatoes and onions in northern Niger. Survey participants reported that the increased profitability of cash crop production has led to the neglect of animal-sourced food production. With the income generated from cash crop sales, rural households purchase imported staple foods, such as rice and wheat products (especially pasta), to supplement locally grown crops. It remains unclear how increased market sales affect diet diversity.^43^ However, our results support the conclusions drawn in other studies^44^ that cash crop production and improved market access can contribute to reducing hunger. In peri-urban areas of Accra, food access and security are particularly constrained by insufficient purchasing power.^45^^,^^46^ The increase in the share of Accra’s peri-urban residents reportedly facing hunger underlines the potentially negative consequences of urban growth and limited off-farm employment opportunities for former farmers. These developments have also been observed in other recent studies on African peri-urban poverty and food insecurity.^47^^,^^48^ Our results support findings of studies in other African countries that urban populations have gained access to more diverse foods available at different food access points (supermarkets, local shops, and open street markets) over the past two decades and are therefore able to choose between different options and diets.^49^ Local shops and open street markets seem to remain the main food access point for residents in all selected study locations, but an increasing share of the urban residents is purchasing in supermarkets. Although only a few supermarkets have been established in northern Niger to date, the proportion of people in urban Agadez purchasing food from them has increased. This aligns with several studies on the growing role of supermarkets in sub-Saharan Africa^50^^,^^51^^,^^52^ and the strong persistence of traditional, largely informal markets as main food access points.^46^^,^^49^

Our findings emphasize the importance of acknowledging complexity and context when addressing land use and food insecurity in West Africa. The identified location-specific differences in our study highlight the need for targeted interventions to support effective food and land system planning. A better understanding is needed of how rising intra-regional trade contributes to increasingly telecoupled land and food systems. For example, as agricultural land in urbanizing Accra is converted to infrastructure, food production is likely displaced to more distant regions. These regions become increasingly connected to urban centers through multidirectional trade of resources. The magnitude of these linkages, however, remains unknown. Declining self-sufficiency due to rising market orientation is making livelihoods increasingly dependent on both natural resource availability for intensified production and on markets, including price dynamics. Policies should therefore focus on promoting sustainable resource use and improving market access through infrastructure investments in agricultural production regions, such as rural Niger. In regions affected by urban growth and cropland loss, such as rural and peri-urban Accra, efforts should prioritize the creation of off-farm employment opportunities and ensuring affordable food prices.

### Limitations of the study

While the selected locations in Niger and Ghana are representative of regional processes of change, our findings are limited in scope. In Niger, we observed a general trend toward expanded market-oriented crop production despite urban built-up growth, whereas, in Ghana, the patterns tend to be more location specific. The studied rural location in Ghana is relatively close to Accra and is thus likely more influenced by built-up growth and cropland loss than other rural areas in the country. Some other rural regions in Ghana experience widespread expansion of cocoa^53^ or cashew production.^54^

The remote sensing analyses are based on manual classifications of cropland from current and past satellite images (approx. 15 years ago) with a resolution of less than 2 m. The slightly lower resolution in the older images may have caused inaccuracies in field-size classifications. Ground truthing was conducted only in the Agadez region of Niger. Therefore, detailed information on past and current cultivated crops in all selected locations could not be included in this study. The selected 50 km^2^ areas are relatively small but, in our view, representative of the locations. They allow for more precise detection of changes in cropland than broader land use studies that rely on lower-resolution satellite imagery (e.g., Landsat at 30 m per pixel). As high-resolution multispectral imagery is not publicly available,^55^ many smallholder farms, often smaller than 1 ha, are not accurately represented in such studies.

Data on intra-regional and international trade for selected food products were obtained from FAOSTAT. Due to high informality levels in the agri-food sector and limited availability of official data,^17^ the results may have some unknown inaccuracies.^15^ While the dataset is limited and contains unavoidable inaccuracies, it is useful for identifying trends. Data on regional-level product trade would be highly valuable for a better understanding of urban-rural linkages; however, such data are unfortunately unavailable.

The household surveys were conducted once with each respondent and do not constitute long-term studies. The analyses are therefore based on respondents’ answers at that specific point in time, reflecting the prevailing social-economic conditions. The assessments of changes in food consumption through questions on dietary habits 20 years in the past are likely subject to recall bias.^56^ Moreover, the analysis did not consider income disparities among the groups surveyed.

The study explores how selected rural-urban locations differ in terms of cropland changes and shifts in food consumption patterns and considers potential dependencies arising from trade. It should be noted, however, that many other factors, including political and economic circumstances, also affect food access and consumption, which we did not address in this study.

## Resource availability

### Lead contact

Requests for further information and resources should be directed to and will be fulfilled by the lead contact, Kira Fastner (kira.fastner@uni-kassel.de).

### Materials availability

This study did not generate new unique reagents.

### Data and code availability

• Land use classification (shapefile) data and survey data reported in this paper will be shared by the lead contact upon request.
• This paper does not report original code.
• Any additional information required to reanalyze the data reported in this paper is available from the lead contact upon request.

## STAR★Methods

### Key resources table

**Table:** 

REAGENT or RESOURCE	SOURCE	IDENTIFIER
**Deposited data**		

Google Earth Images	Maxar Technologies CNES/Airbus	https://earth.google.com
FAOSTAT Data Production FAOSTAT Data Trade	FAOSTAT	https://www.fao.org/faostat/en/#data
Household survey on food access and consumption	This paper	S1, upon request

**Software and algorithms**		

QGIS	QGIS vers. 3.28	https://qgis.org/
ArcGIS Pro	ArcGIS Pro vers. 3.5.2	https://pro.arcgis.com/
Microsoft Excel	Microsoft 365	https://www.microsoft.com/
KoboToolbox, KoboCollect	Kobotoolbox	https://www.kobotoolbox.org/

### Experimental model and study participant details

At each selected study location, at least 50 residents aged older than 40 years were randomly selected to participate in semi-structured surveys on past and present food consumption patterns and dietary habits (Table S1). In total, 430 people were interviewed, of which 33% were female and 67% male, with an average age of 51 years. The survey was set up with KoboToolbox and answers were recorded with the mobile application KoboCollect. The surveys were conducted between November 2023 and August 2024. All participants were interviewed in their homes or on the street, after their informed consent was sought, by trained local surveyors. The survey comprised questions about the current (last two weeks) and past (20 years ago) number of daily meals and snacks, the percentage of staple foods, vegetables, and fruits in the daily diets. Moreover, the frequencies of milk, meat product consumption, and hunger concerns have been recorded. Additionally, the people were asked where food is usually bought. Questions on household income were not included in the survey due to the sensitivity of the information. In all selected rural and peri-urban locations of Niger and Ghana, as well as in urban Agadez, agriculture constitutes the primary source of income for the majority of people. Respondents in urban Niamey had diverse income sources, from informal commerce, agriculture, or the service sector. The selected urban location in Ghana (East Legon) is one of Accra’s wealthiest neighborhoods and residents are not involved in agricultural activities.

The data collection procedure and subsequent use of the data were approved by the Central Ethics Committee of the University of Kassel, Germany.

### Method details

**Figure 6:**
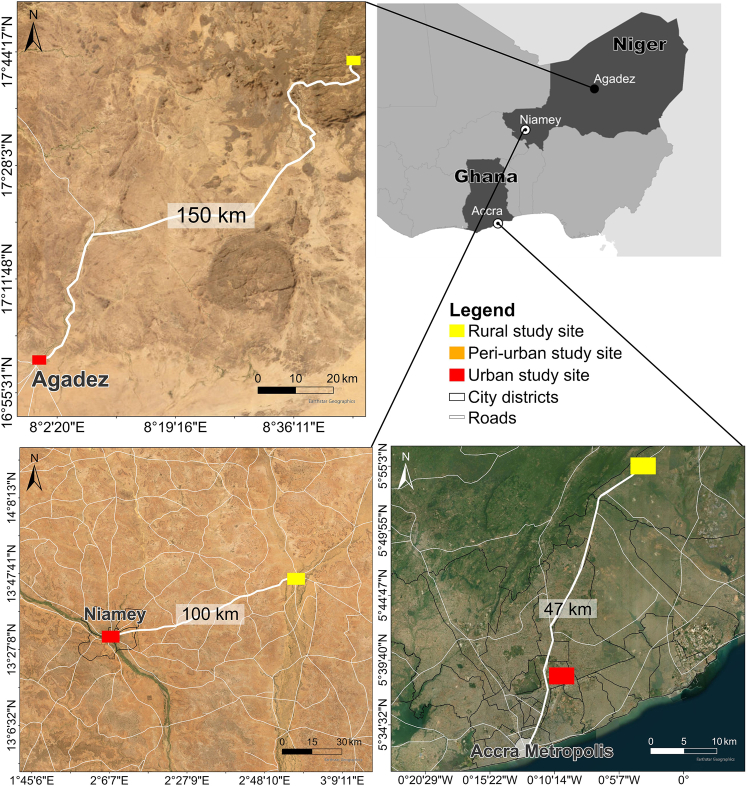
Road networks and selected rural-urban study locations in Agadez and Niamey (Niger) and Accra (Ghana)

We have chosen a rural-urban transect in Ghana’s coastal region and two transects in landlocked Niger in order to identify similarities and differences in food and land systems of the two countries in the same sub-region. The selected rural locations are connected to the cities by road, and there is a regular flow of goods and people. Moreover, the selected urban locations are located along the intra-regional onion transport route, where intensified multidirectional food trading takes place between the countries. Each selected study location covers an area of 50 km^2^ (Figure 6) and is situated along a rural-urban transect following main roads to ensure interconnectivity in land use and food trade, rather than adhering to straight transects based on celestial directions, as commonly applied in other studies.^47^^,^^57^

The rural locations have different spatial distances to the nearest urban centers. In the Agadez region, rural locations more than 150 km away from the city of Agadez (∼173.000 inhabitants in 2025;^58^) were selected. The Agadez region is sparsely populated, infrastructure is poorly developed and further weakened by banditry and political conflict, creating significant challenges for large-scale food transportation and trade. In Niamey, a village (Balléyara) located 100 km away from the urban center was chosen. Niamey’s population grew from 700,000 people in 2000 to 1.6 million in 2025. Southern Niger has a much better developed road infrastructure than the country’s north and is characterized by a few scattered compact urban agglomerations.^4^ In the Greater Accra Region, a rural location (in transition to peri-urban) 47 km away from the urban center was chosen. Given the region’s large urban size with more than 5 million people^59^ and extensive urban peripheries, we also included a peri-urban location 30 km from the urban center in our analysis. Peri-urban regions, in contrast to rural and urban settings, are dynamic landscapes characterized by transitions from non-urban to urban land and exhibit higher levels of socio-economic diversity.^60^ In addition to spatial distances, the classification of our study locations into rural, peri-urban, and urban is therefore based on factors such as land use dynamics, population size, building density, and the predominance of agriculture or non-agriculture as the main livelihood strategy.^1^ This approach is used due to the lack of a universally recognized definition of rural, peri-urban, and urban regions.^1^^,^^60^

For each selected study location, one satellite image from the past, around the year 2010, as well as a recent satellite image, after the year 2023 (depending on availability), were downloaded from Google Earth Pro (Table S2). The high resolution (<2 m) of the RGB-images and the availability of historic images (15–20 years in the past) made it possible to identify changes in cropland. The images were classified with two classes, ‘cropland’ and ‘other’. The boundaries of croplands were identified and mapped from visible concrete, stone, tree or brush fences around fields. The average size of individual classified fields in rural and urban Agadez and in rural Niamey could thus be determined. This allowed us to assess whether the expansion of cropland was accompanied by an increase in the size of individual fields. In urban Niamey and at the locations in Ghana, no individual fields were classified, only total cropland, since individual plots were not clearly identifiable and no cropland expansion occurred. The class ‘other’ includes built-up areas, infrastructure, rivers, forests, and natural vegetation. In both rural and urban environments only clearly distinguishable fields were defined as cropland, while single trees were classified as ‘other’.

Harvested area, import, and export volumes of Niger and Ghana’s main non-animal food products from 2000 to 2023 were retrieved from FAOSTAT^15^ to show changes and volatility in agricultural land use and trade volumes.

### Quantification and statistical analysis

Land use classifications (Figure 2) were carried out in QGIS vers. 3.28 and ArcGIS Pro vers. 3.5 for all selected images by manually digitizing field boundaries. For the calculation of cropland changes, the area of cropland in the classified recent satellite image (2023/2024/2025) was subtracted from the cropland area in the classified image from the past (2008/2009/2010).

Annual totals of the selected FAOSTAT data were calculated and displayed graphically using Microsoft Excel (Figure 3). The compound annual growth rate (CAGR) was calculated, which reflects the geometric mean of year-to-year growth factors. The CAGR was used to capture the growth trends between 2000 and 2023, but it does not account for annual variability.

The survey data was analyzed descriptively using Microsoft Excel (Figures 4 and 5). A change in consumption patterns over the past 20 years (Table 1) was defined based on reported changes by more than half of the respondents at each study location. For example, if more than 50% of surveyed rural Niamey residents reported an increase in their daily vegetable consumption from 0% to 25%, we considered this an overall increase in vegetable consumption for rural Niamey.

## Acknowledgments

We are thankful for the cooperation and trust of the survey participants in the Agadez region, Balléyara, Niamey, and Accra. We thank Mr. Issoufou Issaka, Mr. Salouhou Djibrilla, and Mr. Abdoulkader Ibrahim Mohamed for collecting survey data in areas of Niger that are increasingly difficult for foreigners to access. The 10.13039/501100012687University of Kassel, Germany, provided generous funding and issued academic research and travel permits, even under difficult security conditions. We would like to thank the anonymous reviewer who helped to strengthen the arguments presented in this paper.

## Author contributions

Conceptualization, data curation, formal analysis, methodology, visualization, and writing – original draft: K.F.; investigation and validation: K.F. and K.Y.A.; writing – review and editing: K.Y.A. and A.B.; funding acquisition and resources: A.B.

## Declaration of interests

The authors declare no competing interests.

## References

[1] UN (2018). World Urbanization Prospects. https://population.un.org/wup/.

[2] Gao J., O’Neill B.C. (2020). Mapping global urban land for the 21st century with data-driven simulations and Shared Socioeconomic Pathways. Nat. Commun..

[3] Herrmann S.M., Brandt M., Rasmussen K., Fensholt R. (2020). Accelerating land cover change in West Africa over four decades as population pressure increased. Commun. Earth Environ..

[4] OECD/SWAC (2024). Africapolis (database). http://www.africapolis.org.

[5] Asabere S.B., Acheampong R.A., Ashiagbor G., Beckers S.C., Keck M., Erasmi S., Schanze J., Sauer D. (2020). Urbanization, land use transformation and spatio-environmental impacts: Analyses of trends and implications in major metropolitan regions of Ghana. Land Use Policy.

[6] Behnisch M., Krüger T., Jaeger J.A.G. (2022). Rapid rise in urban sprawl: Global hotspots and trends since 1990. PLOS Sustain. Transform..

[7] Brink A.B., Eva H.D. (2009). Monitoring 25 years of land cover change dynamics in Africa: A sample based remote sensing approach. Appl. Geogr..

[8] De Vos K., Janssens C., Jacobs L., Campforts B., Boere E., Kozicka M., Leclère D., Havlík P., Hemerijckx L.-M., Van Rompaey A. (2024). African food system and biodiversity mainly affected by urbanization via dietary shifts. Nat. Sustain..

[9] Haberman D., Bennett E.M. (2019). Ecosystem service bundles in global hinterlands. Environ. Res. Lett..

[10] Karg H., Drechsel P., Akoto-Danso E., Glaser R., Nyarko G., Buerkert A. (2016). Foodsheds and City Region Food Systems in Two West African Cities. Sustainability.

[11] Karg H., Bouscarat J., Akoto-Danso E.K., Heinrigs P., Drechsel P., Amprako L., Buerkert A. (2022). Food Flows and the Roles of Cities in West African Food Distribution Networks. Front. Sustain. Food Syst..

[12] Kennedy E., Reardon T. (1994). Shift to non-traditional grains in the diets of East and West Africa: role of women’s opportunity cost of time. Food Policy.

[13] Zhou Y., Staatz J. (2016). Projected demand and supply for various foods in West Africa: Implications for investments and food policy. Food Policy.

[14] Ragasa C., Andam K.S., Asante S.B., Amewu S. (2020). Can local products compete against imports in West Africa? Supply- and demand-side perspectives on chicken, rice, and tilapia in Ghana. Glob. Food Sec..

[15] FAOSTAT (2024). Trade: Detailed trade matrix. https://www.fao.org/faostat/en/#data/TM.

[16] Reardon T., Liverpool-Tasie L.S.O., Belton B., Dolislager M., Minten B., Popkin B., Vos R. (2024). African domestic supply booms in value chains of fruits, vegetables, and animal products fueled by spontaneous clusters of SMEs. Appl. Econ. Perspect. Policy.

[17] OECD/SWAC (2025).

[18] Fastner K., Djibrilla S., Nguyen T.T., Buerkert A. (2023). Telecoupled urban demand from West African cities causes social-ecological land use transformation in Saharan oases. PLoS One.

[19] Fastner K., Ibrahim Mohamed A.K., Buerkert A. (2025). Natural resource governance in Niger’s telecoupled oasis systems. Land Use Policy.

[20] Weather and climate (2025). Agadez, Niger Climate. https://weatherandclimate.com/niger/agadez.

[21] L’Hôte Y., Mahé G., Somé B., Triboulet J.P. (2002). Analysis of a Sahelian annual rainfall index from 1896 to 2000; the drought continues. Hydrol. Sci. J..

[22] Ministry of Food and Agriculture Ghana (2022). Statistics Research and Information Directorate, Accra, Ghana.

[23] The World Bank (2025). GDP per capita (current US$) - Ghana, Niger. https://data.worldbank.org/indicator/NY.GDP.PCAP.CD?locations=GH-NE.

[24] The World Bank (2025). Population growth (annual %) - Ghana, Niger. https://data.worldbank.org/indicator/SP.POP.GROW?locations=GH-NE.

[25] International Labour Organization (2024). ILOSTAT database, ILO modelled estimates database. https://ilostat.ilo.org/data/.

[26] Young A. (2013). Inequality, the Urban-Rural Gap, and Migration∗. Q. J. Econ..

[27] Thériault V., Bouscarat J., Heinrigs P., Aparisi A.M., Assima A. (2024). West African Papers, No. 45.

[28] Allen T., Heinrigs P., Heo I. (2018). West African Papers, No. 14.

[29] Oppong J., Namwamba J.B., Twumasi Y.A., Ning Z.H., Asare-Ansah A.B., Akinrinwoye C., Antwi R., Osimbo B.M., Loh P., Frimpong D.B. (2025). Urbanization and urban forest loss: a spatial analysis of five metropolitan districts in Ghana. Geology, Ecology, and Landscapes.

[30] Nasser I.A., Adam E. (2024). Urbanisation in Sub-Saharan Cities and the Implications for Urban Agriculture: Evidence-Based Remote Sensing from Niamey, Niger. Urban Sci..

[31] Bren d’Amour C., Reitsma F., Baiocchi G., Barthel S., Güneralp B., Erb K.-H., Haberl H., Creutzig F., Seto K.C. (2017). Future urban land expansion and implications for global croplands. Proc. Natl. Acad. Sci. USA.

[32] Doe B., Amoako C., Adamtey R. (2022). Spatial expansion and patterns of land use/land cover changes around Accra, Ghana – Emerging insights from Awutu Senya East Municipal Area. Land Use Policy.

[33] Denison J. (2021). Framework for Irrigation Development and Agricultural Water Management in Africa. https://coilink.org/20.500.12592/bppsf6.

[34] Kafle K., Balasubramanya S. (2022). Reducing food insecurity through equitable investments in irrigation: The case of Niger. J. Agr. App. Econ. Assoc..

[35] FAO (2025). Enhancing crop production in the Sahel through innovative technologies in small-scale irrigation systems. Science, Technology and Innovation.

[36] Abdallah A.-H., Ayamga M., Awuni J.A. (2023). Impact of land grabbing on food security: evidence from Ghana. Environ. Dev. Sustain..

[37] Enete A.A., Achike A.I. (2008). Urban Agriculture and Urban Food Insecurity/Poverty in Nigeria. Outlook Agric..

[38] Lee-Smith D. (2010). Cities feeding people: an update on urban agriculture in equatorial Africa. Environ. Urban.

[39] Azunre G.A., Amponsah O., Peprah C., Takyi S.A., Braimah I. (2019). A review of the role of urban agriculture in the sustainable city discourse. Cities.

[40] Puppim de Oliveira J.A., Ahmed A. (2021). Governance of urban agriculture in African cities: Gaps and opportunities for innovation in Accra, Ghana. J. Clean. Prod..

[41] Omotoso A.B., Letsoalo S., Olagunju K.O., Tshwene C.S., Omotayo A.O. (2023). Climate change and variability in sub-Saharan Africa: A systematic review of trends and impacts on agriculture. J. Clean. Prod..

[42] Dzanku F.M., Liverpool-Tasie L.S.O., Reardon T. (2024). The importance and determinants of purchases in rural food consumption in Africa: Implications for food security strategies. Glob. Food Sec..

[43] Nandi R., Nedumaran S., Ravula P. (2021). The interplay between food market access and farm household dietary diversity in low and middle income countries: A systematic review of literature. Glob. Food Sec..

[44] Usman M.A., Haile M.G. (2022). Market access, household dietary diversity and food security: Evidence from Eastern Africa. Food Policy.

[45] Demmler K.M., Ecker O., Qaim M. (2018). Supermarket Shopping and Nutritional Outcomes: A Panel Data Analysis for Urban Kenya. World Dev..

[46] Wanyama R., Gödecke T., Chege C.G.K., Qaim M. (2019). How important are supermarkets for the diets of the urban poor in Africa?. Food Secur..

[47] Chagomoka T., Drescher A., Glaser R., Marschner B., Schlesinger J., Abizari A.-R., Karg H., Nyandoro G. (2018). Urban and peri-urban agriculture and its implication on food and nutrition insecurity in northern Ghana: a socio-spatial analysis along the urban–rural continuum. Popul. Environ..

[48] Haile Aboye B., Gebre-Egziabher T., Kebede B. (2024). Peri-urban food insecurity and coping strategies among farm households in the face of rapid urbanization in Sub-Saharan Africa: Evidence from Ethiopia. Research in Globalization.

[49] Hannah C., Davies J., Green R., Zimmer A., Anderson P., Battersby J., Baylis K., Joshi N., Evans T.P. (2022). Persistence of open-air markets in the food systems of Africa’s secondary cities. Cities.

[50] Reardon T., Timmer C.P., Barrett C.B., Berdegué J. (2003). The Rise of Supermarkets in Africa, Asia, and Latin America. Am. J. Agric. Econ..

[51] Weatherspoon D.D., Reardon T. (2003). The Rise of Supermarkets in Africa: Implications for Agrifood Systems and the Rural Poor. Dev. Policy Rev..

[52] das Nair R. (2020). Handbook on Urban Food Security in the Global South.

[53] Ajagun E.O., Ashiagbor G., Asante W.A., Gyampoh B.A., Obirikorang K.A., Acheampong E. (2022). Cocoa eats the food: expansion of cocoa into food croplands in the Juabeso District, Ghana. Food Secur..

[54] Ashiagbor G., Asare-Ansah A.O., Laari P.B., Asante W.A. (2022). Cashew expansion holds potential for carbon stocks enhancement in the forest-savannah transitional zone of Ghana. Land Use Policy.

[55] Rufin P., Meyfroidt P., Akinyemi F.O., Estes L., Ibrahim E.S., Jain M., Kerner H., Lisboa S.N., Lobell D., Nakalembe C. (2025). To enhance sustainable development goal research, open up commercial satellite image archives. Proc. Natl. Acad. Sci. USA.

[56] Backiny-Yetna P., Steele D., Yacoubou Djima I. (2017). The impact of household food consumption data collection methods on poverty and inequality measures in Niger. Food Policy.

[57] Hoffmann E.M., Schareika N., Dittrich C., Schlecht E., Sauer D., Buerkert A. (2023). Rurbanity: a concept for the interdisciplinary study of rural–urban transformation. Sustain. Sci..

[58] Brinkhoff T. (2020). City Population Niger. http://www.citypopulation.de/en/niger/cities/.

[59] Ghana Statistical Service (2021). Population of Regions and Districts. Ghana 2021 Population and Housing Census, General Report 3A. https://statsghana.gov.gh/gssmain/fileUpload/pressrelease/2021%20PHC%20General%20Report%20Vol%203A_Population%20of%20Regions%20and%20Districts_181121.pdf.

[60] Karg H., Hologa R., Schlesinger J., Drescher A., Kranjac-Berisavljevic G., Glaser R. (2019). Classifying and mapping periurban areas of rapidly growing medium-sized Sub-Saharan African cities: a multi-method approach applied to Tamale, Ghana. Land.

