# Supplementary material for: Rural-urban land and food system changes in West Africa
Source: iScience. 2025 Nov 12;28(12):114007. doi: 10.1016/j.isci.2025.114007 (PMC12688667; doi:10.1016/j.isci.2025.114007)
Supplement: Document S1. Figures S1–S4 and Tables S1–S5 [file mmc1.pdf]

iScience, Volume *28*

Supplemental information

Rural-urban land and food system changes in West Africa

Kira Fastner, Kofi Yeboah Asare, and Andreas Buerkert

Supplemental information

Document S1. Tables S1 and S2.

Table S1. Survey on (past-present) food consumption and dietary habits (only persons over 40 years of age). Related to STAR Methods.

Section 1: General questions

1. Hometown: ________________________ Birthplace: ______________________________
2. Age:____ Number of siblings:_____ Number of children:_____
3. Gender: Male ☐ Female ☐ Other ☐

Section 2: Food consumption in the past (more than 20 years ago (before the year 2000)) as a young adult

4.<br>Did your diet change compared to your time as a young adult?	Yes ☐ (continue with question 5)<br>No ☐ (move to section 3)			
5.<br>How many proper meals did you have as a young adult?	1 meal per day ☐<br>2 meals per day ☐<br>3 meals per day ☐<br>4 meals per day ☐<br>5 meals per day ☐<br>6 meals per day ☐	5a.<br>How many snacks/highly processed foods did you have per day?	1 snack per day ☐<br>2 snacks per day ☐<br>3 snacks per day ☐<br>4 snacks per day ☐<br>5 snacks per day ☐<br>6 snacks per day ☐	5b.<br>What kind of snacks? (nuts, chips, bread, chocolate, cookies, etc.)
6.<br>How much % of your daily food were staple food crops (potato, rice, wheat, maize, millet, etc.)?	0 – 25 % ☐<br>25 – 50 % ☐<br>50 – 75 % ☐<br>75 – 100 % ☐	6a.<br>What was the main staple food?		
7.<br>How much % of your daily food were vegetables (cabbage, carrots, tomatoes, etc.)?	0 – 25 % ☐<br>25 – 50 % ☐<br>50 – 75 % ☐<br>75 – 100 % ☐	7a.<br>What was the main vegetable?		
8.<br>How much % of your daily food were fresh fruits?	0 – 25 % ☐<br>25 – 50 % ☐<br>50 – 75 % ☐<br>75 – 100 % ☐	8a.<br>What was the main fruit?		
9.<br>How often did you consume meat?	Every day ☐<br>More than once a week ☐<br>Once a week ☐<br>Once a month ☐<br>Less than 10 times a year ☐<br>Never ☐	9a.<br>What kind of meat did you eat usually?		
10.<br>How often did you consume milk products (milk, cheese, yoghurt, etc.)?	Every day ☐<br>More than once a week ☐<br>Once a week ☐<br>Once a month ☐<br>Less than 10 times a year ☐<br>Never ☐	10a.<br>What kind of milk products did you usually eat/drink?		

11.<br>How often did you consume food away from home (restaurants, etc.)?	Every day ☐<br>More than once a week ☐<br>Once a week ☐<br>Once a month ☐<br>Less than 10 times a year ☐<br>Once a year ☐<br>Never ☐			
12.<br>What was your favorite food?<br>(If local specialty, please specify ingredients)				
13.<br>Did you worry that you would not have enough food?	Yes, often (more than once a week) ☐<br>Sometimes (once a week or less) ☐<br>Never ☐			
14.<br>How much money did you spend on food per day (only for yourself)?		14a.<br>What was most expensive?		
15.<br>Where did you usually buy your food?	I didn't buy food (only own production) ☐<br>Neighbor ☐<br>Local shop ☐<br>Street market ☐<br>Supermarket ☐			

Section 3: Current food consumption (in the last 30 days)

16.<br>How many meals do you have per day?	1 meal per day ☐<br>2 meals per day ☐<br>3 meals per day ☐<br>4 meals per day ☐<br>5 meals per day ☐<br>6 meals per day ☐	16a.<br>How many snacks/highly processed foods do you have per day?	1 snack per day ☐<br>2 snacks per day ☐<br>3 snacks per day ☐<br>4 snacks per day ☐<br>5 snacks per day ☐<br>6 snacks per day ☐	16b.<br>What kind of snacks?<br>(nuts, chips, bread, chocolate, cookies, etc.)
17.<br>How much % of your daily food are staple food crops (potato, rice, wheat, maize, millet, etc.)?	0 – 25 % ☐<br>25 – 50 % ☐<br>50 – 75 % ☐<br>75 – 100 % ☐	17a.<br>What is the main staple food?		
18.<br>How much % of your daily food are vegetables (cabbage, carrots, tomatoes, etc.)?	0 – 25 % ☐<br>25 – 50 % ☐<br>50 – 75 % ☐<br>75 – 100 % ☐	18a.<br>What is the main vegetable?		
19.<br>How much % of your daily food are fresh fruits?	0 – 25 % ☐<br>25 – 50 % ☐<br>50 – 75 % ☐<br>75 – 100 % ☐	19a.<br>What is the main fruit?		
20.<br>How often do you consume meat?	Every day ☐<br>More than once a week ☐<br>Once a week ☐	20a.<br>What kind of meat do you eat usually?		

	Once a month ☐<br>Less than 10 times a year ☐<br>Never ☐			
21.<br>How often do you consume milk products (milk, cheese, yoghurt, etc.)?	Every day ☐<br>More than once a week ☐<br>Once a week ☐<br>Once a month ☐<br>Less than 10 times a year ☐<br>Never ☐	21a.<br>What kind of milk products do you usually eat/drink?		
22.<br>How often do you consume food away from home (restaurants, etc.)?	Every day ☐<br>More than once a week ☐<br>Once a week ☐<br>Once a month ☐<br>Less than 10 times per year ☐<br>Once a year ☐<br>Never ☐			
23.<br>What is your favorite food?<br>(If local specialty, please specify ingredients)				
24.<br>Do you worry that you do not have enough food? (in the last 30 days)	Yes, often (more than once a week) ☐<br>Sometimes (once a week or less) ☐<br>Never ☐			
25.<br>How much money do you spend on food per day (only for yourself)?		25a.<br>What is most expensive?		
26.<br>Where do you usually buy food?	I don’t buy food (only own production) ☐<br>Local shop ☐<br>Farmer’s market ☐<br>Supermarket (in city) ☐			

Table S2. Selected study locations with names of towns/districts and respective satellite image sources for remote sensing analyses. Related to STAR Methods.

Study location (Name of town/district)	Date (YYYY-MM-DD)	Sensor/Image
Rural Agadez *(Mont Bagzam)*	2010-10-22<br>2025-05-10	Maxar Technologies<br>CNES / Airbus
Urban Agadez (*City center*)	2009-12-22<br>2025-03-14	Maxar Technologies<br>Airbus
Rural Niamey *(Balléyara)*	2010-01-04<br>2023-11-07	CNES / Airbus<br>Maxar Technologies
Urban Niamey (*City center*)	2008-02-13<br>2025-05-04	Maxar Technologies<br>Airbus
Rural Accra (*Ayikuma)*	2009-11-22<br>2025-02-22	CNES / Airbus<br>CNES / Airbus
Peri-urban Accra *(Danfa, Kweiman)*	2008-02-18<br>2025-04-10	Maxar Technologies<br>Maxar Technologies
Urban Accra *(City center - East Legon)*	2008-02-18<br>2024-11-24	Maxar Technologies<br>Maxar Technologies
